# Beliefs and Attitudes of Medical Students from Public and Private Universities in Malaysia towards Individuals with HIV/AIDS

**DOI:** 10.1155/2013/462826

**Published:** 2013-10-29

**Authors:** Koh Kwee Choy, Teh Jae Rene, Saad Ahmed Khan

**Affiliations:** ^1^Department of Medicine, Clinical School, International Medical University, Jalan Rasah, 70300 Seremban, Malaysia; ^2^Department of Pharmacy, International Medical University, Plaza Komanwel, Bukit Jalil, 57000 Kuala Lumpur, Malaysia; ^3^Department of Restorative Dentistry, Faculty of Dentistry, University of Malaya, 50603 Kuala Lumpur, Malaysia

## Abstract

We describe the findings from a survey assessing the beliefs regarding testing, confidentiality, disclosure, and environment of care and attitudes towards care of people with HIV/AIDS (PLHWA), in 1020, 4th and 5th year medical students, from public and private medical universities in Malaysia. A self-administered validated questionnaire based on the UNAIDS Model Questionnaire with a 5-point Likert scale (5, strongly disagree; 4, disagree; 3, neutral; 2, agree; 1, strongly agree) was used as a survey tool. The survey included demographic data and data on undergraduate training received on HIV/AIDS. Statistical significance in the demographic data and training received by respondents was evaluated using the chi-square test while the independent Student's *t*-test was used for comparison of means between public and private universities. A *P* value of <0.05 was considered statistically significant with 95% confidence interval. Our study revealed less than 20% of medical students received adequate training to care for PLHWA. They had prevalent negative beliefs regarding testing, confidentiality, disclosure and environment of care towards PLHWA although in giving care to PLHWA, their attitudes were largely positive and nondiscriminatory.

## 1. Introduction

The first few cases of AIDS in Malaysia were diagnosed between 1986 and 1987. Since then the number of reported cases has increased at an alarming rate. By the end of the year 2011, there were 94,841 cases of HIV infection and 17,686 cases of Acquired Immune Deficiency Syndrome (AIDS) reported in Malaysia with estimated 79, 855 people living with HIV/AIDS (PLWHA) [1]. The main modes of transmission of Human Immunodeficiency Virus (HIV) in Malaysia are the sharing of contaminated needles through intravenous drug use and heterosexual transmission. Highly Active Antiretroviral Therapy (HAART) was first made available in 1989 [2].

Stigma and discrimination continue to be major limiting factors in the care and prevention of HIV/AIDS particularly in the healthcare sector with reports documenting up to a third of PLHWA who refused care by healthcare providers in some countries [3, 4]. In Malaysia, narrative reports have identified stigma and discrimination as barriers in the prevention, treatment, and provision of care to PLHWA [5]. 

Negative attitudes in clinicians towards PLWHA manifested through practices such as charting, isolation, breach of confidentiality, refusal to care, and testing without consent have been documented. These negative attitudes were attributed to inadequate knowledge, fear, and misconceptions regarding HIV/AIDS [6–8]. Several studies have shown that knowledge regarding HIV/AIDS, its transmission, and treatment among university students have a significant effect in reducing prejudicial attitudes towards PLWHA although the change was often observed to be minute or minimal [9–13]. Similarly, studies conducted among medical and pharmacy students in Malaysia have reported lack of knowledge regarding treatment and transmission of HIV/AIDS and discriminatory attitudes towards PLHWA [14, 15].

Belief is defined as “individually held subjective ideas about the nature of the object or an event”, and attitudes are defined as “learned tendencies to act or respond in a specific way to events, people or orientations” [16]. Beliefs and attitudes are closely linked. Culturally instilled beliefs exert a strong influence on attitude [17]. Our study was designed to assess the beliefs and attitudes towards PLHWA among Malaysian medical students in the clinical years of undergraduate training with comparison between students from public and private medical universities.

## 2. Materials and Methods

### 2.1. Study Setting and Sample Size 

This study was a cross-sectional survey conducted from July 2012 to October 2012. One thousand and twenty 4th and 5th year medical students from 7 public and private universities in Malaysia responded to the survey. Sample size required for statistical significance was calculated to be 377 assuming 95% confidence interval (CI) with 5% margin error and 50% response rate.

### 2.2. Survey Tool

The survey tool was a self-administered questionnaire adapted from the UNAIDS Model Questionnaire and has been used in studies elsewhere [18, 19]. The survey tool was piloted and validated with 20 randomly chosen 4th and 5th medical students and minor adjustments were made postpiloting mainly to facilitate better comprehension before distribution. The questionnaire contained two parts. The first part comprised of items that explored the demographics and training on the subject of HIV/AIDS received by the respondents. The second part consisted of two domains. The first domain assessed the respondent's beliefs regarding testing, confidentiality, disclosure, and environment of care (eight items) while the second domain assessed the attitudes towards care given to HIV/AIDS patients (nine items). A 5-point Likert scale ranging 5 (strongly disagree), 4 (disagree), 3 (neutral), 2 (agree), and 1 (strongly agree) was used for each item. The maximum scores for the first and second domains were 40 and 45, respectively. 

### 2.3. Data Collection

Data collection was collected by the research team in 7 public and private medical universities in Malaysia. Medical students in their 4th and 5th year of studies in these universities were briefed on the objectives of the survey and participation was voluntary. Informed consent was taken from the respondents before distribution of the survey tool. Respondents were allowed 15 minutes to complete the questionnaire anonymously without interruption at their institution. 

### 2.4. Definition

The abbreviation PLHWA is used in this study to mean either “*people* living with HIV/AIDS” or “*patient *living with HIV/AIDS.” Both terms are interchangeable depending on the context of whether the HIV-infected individual was admitted (*patient*) or otherwise (*people*). 

### 2.5. Ethical Considerations

The International Medical University Research Joint and Ethics Committee approved the study. 

### 2.6. Statistical Analysis

Data was presented in mean or percentage where appropriate. Descriptive statistics were obtained for different quantitative variables. Demographic data and training received by respondents were evaluated using the chi-square test. The independent Student's *t*-test was used to compare the means between public and private universities. Statistical significance was set at *P* < 0.05. All statistical analyses were done using the Statistical Package for Social Sciences (SPSS) for Windows version 20. 

## 3. Results

### 3.1. Demographic Characteristics

The demographic characteristics of the students are shown in Table 1. The male to female ratio was nearly 1 : 2. Two-thirds of the respondents were from private medical universities. The 4th and 5th year students were nearly equally represented. Students from the Malay ethnic group were the largest group followed by the Chinese and Indians.

### 3.2. Training Related to HIV/AIDS

Details regarding training related to HIV/AIDS received by the students are shown in Table 2. Most of the respondents (88.7%) reported having covered HIV/AIDS as a topic in their medical program with no statistical difference between students in the public and private universities (86.8% versus 99.9%, respectively; *P* = 0.138). Both groups reported looking up information about HIV/AIDS from the internet (82.5%) and medical books (73.4%). However, less than half of them (49.3%) reported that they have read literature studies related to HIV/AIDS on their own time with students from the public universities reporting a significantly lower percentage compared to students from private universities (43.7 versus 52.6%; *P* = 0.006). Less than half of the public and private medical students have reviewed HIV/AIDS case studies (41.0% versus 41.4%; *P* = 0.893). Less than one-third of the students have attended conferences on HIV/AIDS (29.9% public versus 27.1% private; *P* = 0.338). Significantly more public medical students have participated in activities/events related to HIV/AIDS topic compared to private medical students (31.2% versus 24.6%; *P* = 0.022).

One-fourth (25.1%) of the students reported ever having provided care to PLHWA whereas only 19.3% of the students reported having received adequate training to provide care to PLHWA (24.3% public versus 25.5% private; *P* = 0.688). More private medical students felt they were effectively trained to provide care to PLHWA compared to public medical students (21.1% private versus 16.1% public; *P* = 0.049), although the total number of students who felt this way was low (19.3%). 

### 3.3. Beliefs Regarding Testing, Confidentiality, Disclosure, and Environment of Care towards PLHWA

The findings in this domain are shown in Table 3. Students from both public and private medical universities disagreed with testing patients for HIV infection without consent. They also disagreed that relatives of PLHWA should be notified of the HIV status without the patient's consent. There was no statistical difference between the two groups of students (*P* > 0.05).

Both public and private medical students agreed that all healthcare workers should be routinely tested for HIV/AIDS; sexual partners of PLWHA should be notified of the HIV status even without the patient's consent; that is, it is the responsibility of healthcare personnel to inform the spouse, partner, boyfriend, or girlfriend of the patient's HIV status, and the charts of PLHWA should be clearly marked, so clinic/hospital workers will know the patient's status. There was no statistical difference between the two groups of students (*P* > 0.05) in all these items.

The two groups of students differed with regard to the issue of routine testing for HIV being part of the admission process for all patients. Public medical students were not in favor while private medical students were in favor of it. However, the difference was not statistically significant (*P* = 0.05). Public medical students disagreed that rooms or beds of PLWHA should be clearly marked so hospital workers will know the patient's status while private medical students were neutral about this issue. There was no statistical difference between the two groups of students (*P* > 0.05). 

### 3.4. Attitudes towards Care Given to PLHWA

The findings from this domain are shown in Table 4. Both public and private medical students were concerned that they were not trained to properly care for PLHWA, although the latter were less concerned compared to the former (*P* = 0.003). Both groups of students were unconcerned about stigmatization from family members or friends for providing care to PLHWA with private medical students being less concerned compared to public medical students (*P* < 0.001). Regarding refusal to treat PLHWA in order to protect self and family, avoidance of care for PLHWA, feeling uncomfortable around PLHWA, and being uncomfortable providing care to family members with HIV/AIDS, both public and private medical students disagreed with these items in the questionnaire with the latter group feeling more strongly about the matters compared to the former (all *P* values < 0.005). 

The public and private medical students differed on 2 items in the questionnaire, namely, being more comfortable providing care to non-HIV patients compared to PLHWA with the former agreeing while the latter disagreeing (mean 2.91 versus 3.13; *P* = 0.002), and the fear of becoming infected with HIV if needed to provide care to PLHWA, again with the former agreeing while the latter disagreeing with the statement (mean 2.99 versus 3.29; *P* < 0.001).

## 4. Discussion

### 4.1. Demography and Training

The gender distribution of the students in our study accurately mirrored the situation in most medical universities in Malaysia, namely, the larger population of female students compared to males. The 4th and 5th year students were well represented in equal proportions as with the distribution by ethnicity. With regard to training related to HIV/AIDS received by these students, most of them reported covering HIV/AIDS as a subject and have sourced for information on HIV/AIDS from either books or the internet although significantly more private students also read current literature or journals related to HIV/AIDS in their own time (Table 2, items 1–4). Knowledge did not always translate to experience as less than half of the students had reviewed HIV/AIDS case studies and even fewer had attended conferences on HIV/AIDS; participated in activities or events related to HIV/AIDS; provided care for PLWHA; or were effectively trained to provide care to PLHWA (Table 2, items 5–9). Medical students in Malaysia generally have limited opportunities to participate in a meaningful way in the various conferences or activities related to HIV/AIDS. Furthermore, PLHWA admitted for various ailments are usually small in number in the wards compared to other disease conditions thus further limiting the exposure of the students to the care of these patients.

### 4.2. Beliefs Regarding Testing, Confidentiality, Disclosure, and Environment of Care towards PLHWA

 Public and private medical students disagreed that HIV test can be done without the knowledge or permission of the patient under certain circumstances (Table 3, item 1). This belief is in keeping with the current practice in Malaysia, which follows the guidelines issued by the World Health Organization (WHO) in 2007 where explicit consent must be obtained from the patient before HIV testing can be done. In critically ill or unconscious patients who may not be able to provide informed consent to HIV testing and counseling, consent should be obtained from the patient's next-of-kin, guardian, or other caregiver. Only in the absence of such a person, healthcare providers should act according to the best interests of the patient concerned [20].

We found out that both public and private medical students agreed that healthcare workers should be routinely tested for HIV (Table 3, item 2), although currently this is not the policy in the Malaysian healthcare system. However, they differed with regard to routine testing for HIV as part of admission process for all patients (Table 3, item 3). Private medical students were supportive of this statement while public medical students differed. 

In 2006, the Centers for Disease Control and Prevention (CDC) issued a statement recommending routine HIV testing for Americans of ages 13–64. The aim was to increase awareness of HIV status in order to reduce the spread of disease transmission. The CDC recommended that patients be informed that HIV testing is a routine part of medical care. Assent is inferred unless specifically declined by patients, also known as opt-out testing [21]. However, opponents to the statement expressed concerns regarding stigma and discrimination associated with routine HIV testing [22, 23]. Regardless, the CDC recommendation also explicitly stated that testing should only be carried out with patients' consent after extensive pre- and post-test counseling have been provided [21]. 

Currently, the Malaysian government has implemented mandatory HIV screening for donated blood, blood products, and organs, routine screening for most-at-risk-populations (MARP), premarital screening for Muslims, and opt-out antenatal screening. Free HIV testing is available in all government health facilities through voluntary, counseling, and testing (VCT) and anonymous HIV testing. The policy of opt-out testing is not widely practiced in all government health facilities [1]. In contrast, some private hospitals in Malaysia have implemented the opt-out testing policy. Compared to their peers in public universities, private medical students in our study may be more aware of this practice and this may have influenced their response with regard to routine testing for HIV as part of admission process for all patients. 

In our study, public and private medical students disagreed that relatives of PLHWA should be notified of the patient's HIV status, even without the patient's permission (Table 3, item 4). This is consistent with the current practice in Malaysia where the HIV status of a patient can only be revealed with the explicit consent of a PLHWA. Of concern is the apparent readiness of public and private medical students to reveal the HIV status of PLWHA to the sexual partners of the patient, even without the latter's permission (Table 3, item 5), clearly breaching the current practice in Malaysia where the legal spouse of the PLHWA may be informed of the HIV status of the patient only if the latter is not willing to reveal the status him/herself and with his/her consent. Several studies among medical students elsewhere have reported similar findings [24–26]. Perhaps the students felt duty bound to warn sexual partners of the patient that may be placed in harm's way which has led to this response. In the Global Medical Ethics Manual, “disclosure to a spouse or current sexual partner of a HIV-positive patient may not be unethical and, indeed, may be justified” but only “when the patient is *unwilling *to inform the person(s) at risk” [27]. 

The students also agreed that healthcare personnel have the responsibility to inform a spouse, partner, boyfriend, or girlfriend the HIV status of a PLWHA (Table 3, item 6). This is against the current practice in Malaysia where the onus is on the PLHWA to inform others regarding his HIV status. The current practice in Malaysia is to give the patient a reasonable amount of time after diagnosis of their infection, usually within 2 weeks, to inform their legally married spouse regarding their disease status. However, there is no provision by law to inform sexual partners who are not legally partners of the patient although this is encouraged.

Unfavorable belief leading to potential for discrimination against PLWHA is revealed in this study where students agreed to the marking of charts in order to disclose patients' HIV status to healthcare workers (Table 3, item 8). Such discriminatory belief was also reported in several studies involving healthcare workers [18, 22]. Intentionally identifying and labeling those infected with HIV is an infringement of the patients' right to autonomy and confidentiality. Moreover, it breaches the duty and responsibility of healthcare providers to uphold the principles of justice, nonmaleficence, and beneficence pledged in the Declaration of Geneva which distinctly stated that “information of patients could only be disclosed to other healthcare providers on a strictly need-to-know basis unless the patient has given explicit consent” [28]. The Namibian HIV-AIDS Charter of Rights also states that “segregation, isolation or quarantine of persons in prisons, schools, hospitals or elsewhere merely on the grounds of HIV/AIDS is unjustified and should therefore not be permitted” [29]. 

Similarly, the students in our study took a neutral stand regarding the marking of rooms or beds of PLHWA in order to disclose their HIV status (Table 3, item 7). The identification of PLHWA by marking their hospital charts or the rooms is not practiced in Malaysia. This is a clear indication of uncertainty of the medical students' ethical duty to uphold the ethical standards of confidentiality of patients. Such beliefs need to be rectified before it manifest as unethical behavior resulting in discriminatory practices when they eventually become doctors.

In summary, the outcome of the survey in this domain revealed relatively prevalent negative or unfavorable beliefs towards PLHWA among medical students. It highlights an urgent need for medical ethics education to be integrated into curriculum of universities. Many medical schools in the West have vigorously incorporated medical ethics into their curriculum [30–33]. Similar vigor is often lacking in medical schools in other parts of the world including Malaysia. In one of the private medical schools surveyed in this study, medical ethics regarding HIV/AIDS were not taught exclusively as a topic in the curriculum.

### 4.3. Attitudes towards Giving Care to Individuals with HIV/AIDS

In contrast to the negative beliefs identified in the first domain, we identified mostly positive attitudes towards PLHWA among the medical students (Table 4). The findings are in stark contrast to reports from several local studies and elsewhere where negative attitudes towards PLHWA were prevalent among medical students and healthcare workers [11–15, 24, 25, 34]. The students in this study reported that they were not trained to properly counsel a patient with HIV/AIDS. In another study done in Malaysia, Samant et al. reported only a minority of final year medical students who felt competent to counsel HIV/AIDS patients [13]. This may be attributable to the lack of clinical exposure in providing care to PLHWA, as only 25.1% of students in this study reported having had the experience. It has been shown that constant clinical encounters with such patients lead to more positive attitude and therefore extensive practical exposure to HIV/AIDS patients during the medical students' clinical years has been recommended [14, 24–26, 35, 36]. 

The students in this study reported being unconcerned about stigmatization by family and friends when caring for PLHWA; they will not refuse to treat PLHWA to protect self and family; they will not avoid caring for PLHWA; they will not feel uncomfortable around PLHWA; and they will not feel uncomfortable providing care for a family member infected with HIV (Table 4, items 2–8). Of interest is the significant difference in the responses between the 2 groups of students, with private medical students being more positive in their attitudes compared to their counterpart in the public medical schools. The positive attitudes displayed by these students are commendable and stand in sharp contrast to the prevalent negative attitudes found in other studies.

On the other hand, we found that students from public universities reported being afraid of becoming infected with HIV while caring for PLHWA and preferred to care for non-HIV patients instead (Table 4, items 4 and 9). Similar findings of such fears and preferences have been documented elsewhere among medical students and healthcare workers [13, 18, 37, 38]. These attitudes have been largely attributed to cases of HIV transmission via needle prick injury sustained during the course of caring for PLHWA [39, 40]. Intervention measures that increase skills of healthcare professionals towards PLHWA have been associated with attitudinal change [41]. Therefore, promotion of universal precautions to medical students in practical experiences should help to address such fear [24]. In comparison, private medical students did not have this distinction between HIV and non-HIV infected patients (*P* = 0.002) and had less fear of being infected with HIV while caring for PLHWA (*P* < 0.001).

There are several limitations in our study. Firstly, the responses gathered using a self-administered questionnaire is subjected to possible bias and the accuracy of the responses could not be readily verified. Secondly, the survey tool was not designed to explore in depth the undergraduate training related to HIV/AIDS received by the students. Future studies should explore this aspect, which may provide more valuable insight into the relationship between training, beliefs, and attitudes. And finally, the survey tool did not explore the socioreligious background of the respondents, living in a multicultural society in Malaysia, which may have influenced their beliefs and attitudes. 

## 5. Conclusions

We found that medical students in Malaysia had prevalent negative beliefs regarding testing, confidentiality, disclosure and environment of care towards PLHWA. However, the attitudes of these students towards giving care to PLHWA were largely positive and nondiscriminatory. We recommend that medical ethics education related to HIV/AIDS be vigorously integrated into current existing Malaysian medical curriculum. We also recommend early and extensive exposure to the care of PLHWA among medical students in Malaysia. 

## Figures and Tables

**Table 1:** Demographic characteristics of survey respondents (*N* = 1020).

Variable	Number (*N*)	Percent (%)
Gender		
Male	378	37.1
Female	642	62.9
University		
Public	378	37.1
Private	642	63.9
Year		
Fourth	505	49.5
Fifth	515	50.5
Ethnicity		
Malay	483	47
Chinese	313	31
Indian	187	18
Others	37	4

**Table 2:** Training related to HIV/AIDS received by survey respondents (*N* = 1020).

No.	Item	Public		Private		Total		*P* value
		*N*	(%)	*N*	(%)	*N*	(%)	
1	I have covered HIV/AIDS as a topic in my program	328	(86.8)	577	(99.9)	905	(88.7)	0.138
2	I have researched Internet sources about HIV/AIDS	311	(82.3)	530	(82.6)	841	(82.5)	0.910
3	I have read books in my own time about HIV/AIDS	282	(74.6)	467	(72.7)	749	(73.4)	0.516
4	I read the current literature/journals on my own time to learn about HIV/AIDS	165	(43.7)	338	(52.6)	503	(49.3)	0.006
5	I have reviewed HIV/AIDS case studies	155	(41)	266	(41.4)	421	(41.3)	0.893
6	I have attended conferences on HIV/AIDS	113	(29.9)	174	(27.1)	287	(28.1)	0.338
7	I have participated in activities/events related to HIV/AIDS topic	118	(31.2)	158	(24.6)	276	(27.1)	0.022
8	I have provided care to someone with HIV/AIDS	92	(24.3)	164	(25.5)	256	(25.1)	0.688
9	I have been effectively trained to provide care to a person with HIV/AIDS	61	(16.1)	136	(21.2)	197	(19.3)	0.049

**Table 3:** Public and private medical students' beliefs regarding testing, confidentiality, disclosure, and environment of care towards PLHWA.

Items	Public*						Private*						*P* value^#^
	1	2	3	4	5	mean	1	2	3	4	5	mean	
(1) There are circumstances where it is appropriate to test a patient for HIV/AIDS without the patient's knowledge or permission	23	88	64	121	82	3.40	51	138	103	176	174	3.44	0.604
(2) All healthcare workers should be routinely tested for HIV/AIDS	101	176	61	34	6	2.12	208	262	93	64	15	2.09	0.631
(3) HIV/AIDS testing should be routinely performed as part of the admission process for all patients	31	85	107	122	33	3.11	92	172	154	160	64	2.89	0.005
(4) Relatives of patients with HIV/AIDS should be notified of the patient's HIV status, even without the patient's permission	27	75	56	149	71	3.43	61	121	102	179	179	3.46	0.724
(5) Sexual partners of patients with HIV/AIDS should be notified of the patient's status, even without the patient's permission	107	172	37	46	16	2.19	197	233	72	84	56	2.33	0.068
(6) Health care personnel have the responsibility to inform a spouse/partner or boyfriend/girlfriend of the patient's HIV status	73	190	53	53	9	2.30	167	270	110	61	34	2.26	0.576
(7) The rooms/beds of patients with HIV/AIDS should be clearly marked so hospital workers will know the patient's status	42	90	76	123	47	3.11	102	151	131	164	94	3.00	0.153
(8) The charts of patients with HIV/AIDS should be clearly marked so clinic/hospital workers will know the patient's status	64	171	66	52	25	2.48	136	272	108	82	42	2.41	0.338

*Number of respondents under each category of the Likert scale: 1: strongly agree; 2: agree; 3: neutral; 4: disagree; 5: strongly disagree.

^#^ *P* value derived from comparison of means between public and private medical students; 95% confidence interval.

**Table 4:** Public and private medical students' attitudes towards giving care to individuals with HIV/AIDS.

Items	Public*						Private*						*P* value^#^
	1	2	3	4	5	mean	1	2	3	4	5	mean	
(1) I am concerned that I am not trained to properly counsel a patient with HIV/AIDS	70	215	68	19	6	2.14	126	284	146	69	17	2.33	0.003
(2) I am concerned about being stigmatized by my family because I have to provide care for patients with HIV/AIDS	6	42	92	165	73	3.68	13	49	133	205	242	3.96	<0.001
(3) I am concerned about being stigmatized by friends because I have to provide care for patients with HIV/AIDS	3	44	78	170	83	3.76	10	43	119	215	255	4.03	<0.001
(4) I will be more comfortable giving care to non-HIV patients than to HIV- positive/AIDS patients	27	114	125	90	22	2.91	34	176	201	132	99	3.13	0.002
(5) I would refuse to treat a patient infected with HIV/AIDS to protect my family and myself	2	11	56	210	99	4.04	4	14	90	263	271	4.22	<0.001
(6) I may try to avoid caring for HIV/AIDS patients	2	24	71	195	86	3.90	6	29	113	254	240	4.08	0.001
(7) I feel uncomfortable around people who are infected with HIV/AIDS	4	47	109	161	57	3.58	10	77	157	218	180	3.75	0.010
(8) I would feel uncomfortable to provide care for a family member sick with HIV/AIDS	1	21	62	210	84	3.94	7	31	99	258	247	4.10	0.004
(9) I fear becoming infected with HIV if I have to care for an HIV/AIDS patient	19	144	79	94	42	2.99	40	155	158	156	133	3.29	<0.001

*Number of respondents under each category of the Likert scale: 1: strongly agree; 2: agree; 3: neutral; 4: disagree; 5: strongly disagree.

^#^ *P* value derived from comparison of means between public and private medical students; 95% confidence interval.
